# Transformation of Environmental *Bacillus subtilis* Isolates by Transiently Inducing Genetic Competence

**DOI:** 10.1371/journal.pone.0009724

**Published:** 2010-03-16

**Authors:** Reindert Nijland, J. Grant Burgess, Jeff Errington, Jan-Willem Veening

**Affiliations:** 1 Dove Marine Laboratory, School of Marine Science and Technology, Newcastle University, North Shields, United Kingdom; 2 Centre for Bacterial Cell Biology, Institute for Cell and Molecular Biosciences, The Medical School, Newcastle University, Newcastle upon Tyne, United Kingdom; University of British Columbia, Canada

## Abstract

Domesticated laboratory strains of *Bacillus subtilis* readily take up and integrate exogenous DNA. In contrast, “wild” ancestors or *Bacillus* strains recently isolated from the environment can only be genetically modified by phage transduction, electroporation or protoplast transformation. Such methods are laborious, have a variable yield or cannot efficiently be used to alter chromosomal DNA. A major disadvantage of using laboratory strains is that they have often lost, or do not display ecologically relevant physiologies such as the ability to form biofilms. Here we present a method that allows genetic transformation by natural competence in several environmental isolates of *B. subtilis*. Competence in these strains was established by expressing the *B. subtilis* competence transcription factor ComK from an IPTG-inducible promoter construct present on an unstable plasmid. This transiently activates expression of the genes required for DNA uptake and recombination in the host strain. After transformation, the *comK* encoding plasmid is lost easily because of its intrinsic instability and the transformed strain returns to its wild state. Using this method, we have successfully generated mutants and introduced foreign DNA into a number of environmental isolates and also *B. subtilis* strain NCIB3610, which is widely used to study biofilm formation. Application of the same method to strains of *B. licheniformis* was unsuccessful. The efficient and rapid approach described here may facilitate genetic studies in a wider array of environmental *B*. *subtilis* strains.

## Introduction

*Bacillus subtilis* is a well-studied Gram-positive bacterium, and a major reason for its establishment as a model organism is its capability to take up DNA and integrate it into its genome. This natural competence allows for easy genetic manipulation and for the molecular dissection of gene regulation and cell biological functions. The “domesticated” laboratory strains were originally manipulated to become strongly naturally competent [1]. Recently, an increasing number of environmental strains of *B. subtilis* have been isolated and characterised. These strains can display a plethora of interesting, ecologically or medically relevant properties that are different from the standard laboratory strains. These include anti-fungal activity, biofilm formation or fruiting body formation [2], [3].

Unfortunately, these “wild” strains are often more difficult to transform using natural competence. A “wild” *B. subtilis* strain that is used by many laboratories to study processes such as biofilm formation is NCIB3610. Since this strain is poorly naturally competent, phage transduction is a commonly used technique to introduce alterations into its genome (see e.g. references [2], [4], [5]). Although phage transduction is efficient, unwanted portions of the wild strain's genome might be replaced by the donor strain [6]. Other methods are available to introduce DNA into these non-naturally competent strains, such as electroporation, protoplast transformation and even protoplast electroporation [3]. However, these methods are generally less effective than using natural competence.

The master regulator of natural competence in *B. subtilis* is ComK [7], [8]. Overexpression of *comK* can induce natural competence and this technique has been used to successfully transform the subtilin producer *B. subtilis* strain ATCC6633 [9] and the food spoiling *Bacillus cereus* strain ATCC14579 [10]. Unfortunately, since ComK is a transcriptional regulator with pleiotropic effects, its overexpression will disturb natural gene expression patterns. Continuous overexpression of ComK can re-wire the expression of the entire genome [11]. Even in the heterologous host *Lactococcus lactis*, the expression of *B. subtilis* ComK altered expression of over 200, or almost 10% of all genes present [12].

To induce natural competence without the negative side effects of ComK overexpression in the resulting mutants, we have designed a method based on temporary introduction of a plasmid containing an inducible copy of *comK*. After introduction of this plasmid, and induction of *comK*, the cells become competent even in nutrient rich media such as LB, a medium that normally does not support competence development. After successful transformation of the host, the plasmid can be removed in a single step, and the resulting modified strain will be returned to its wild state. Using this method we were able to transform the otherwise poorly-competent *B. subtilis* NCIB3610, *B. subtilis* ATCC6633 and a wild *B. subtilis* strain isolated from mid Atlantic ridge sediments. We also attempted to introduce natural competence in a number of *B. licheniformis* strains using the same approach, but were unsuccessful.

## Results and Discussion

### Artificial induction of ComK using pLK induces competence

**Figure 1:**
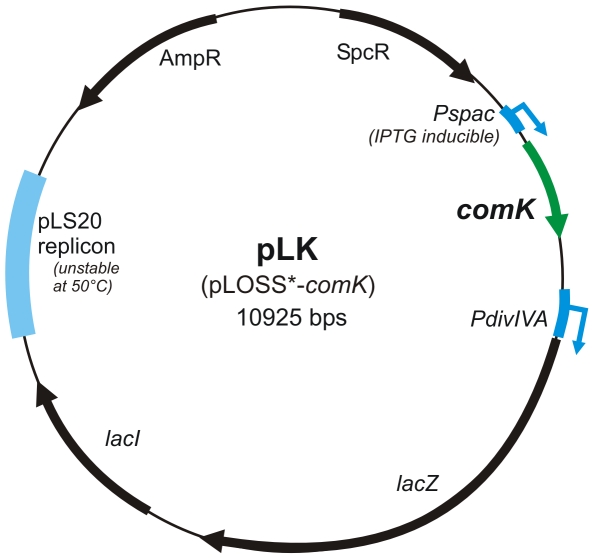
The pLOSS* derived plasmid pLK containing an IPTG inducible *comK* gene. *P_spac_* =  IPTG inducible promoter, *P_divIVA_* =  constitutive *B. subtilis divIVA* promoter used to drive expression of *lacZ*, AmpR  =  ampicillin resistance gene, SpcR  =  spectinomycin resistance gene. Plasmid pLK has been made available through the Bacillus Genetic Stock Centre, with accession number ECE219.

*Bacillus subtilis* 168 is easily made competent by growing it in competence starvation medium. Artificial induction of *comK* expression can further induce competence development in *B. subtilis* 168 [13]. Although wild *B. subtilis* strains often have interesting properties, they are generally less competent for DNA uptake and integration, and therefore more difficult to study using gene transfer methods [9]. Previously, it was shown that constitutive overproduction of ComK can also induce competence in wild *Bacillus* strains [9]. However, constitutive ComK expression might be detrimental to cell growth [14] and potentially rewires global gene transcription [12]. Thus, we set out to construct a system in which *comK* expression could be induced transiently. We constructed the multicopy plasmid pLK, carrying the *B. subtilis comK* gene driven by the IPTG-inducible *P_spac_* promoter, based on the pLOSS* vector (Fig. 1). This multicopy plasmid is intrinsically unstable at higher temperatures and can therefore easily be cured from the host strain [15].

**Figure 2:**
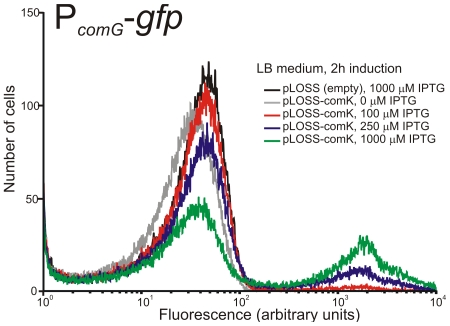
Flow cytometric analysis of 168-ComG-GFP+pLK for induced expression of P*comG*-GFP, 2 hours post induction. Competence induction was observed by measuring individual GFP fluorescence of each cell. At least 85,000 cells were analysed for each sample.

To test if the plasmid could induce the competence regulon we introduced it into *B. subtilis* strain 168-ComG-GFP. This *B. subtilis* 168 derivative contains the promoter of the *comG* operon fused to GFP, a good reporter for identification of cells which have initiated competence development [13]. Strain 168-ComG-GFP without a plasmid, or containing pLOSS* (empty vector) or pLK (*P_spac_*-*comK*), were analysed by flow cytometry under different induction conditions in non-competence inducing LB medium (Fig. 2).

When no IPTG is added and the strain is grown in LB, no difference is observed between wild type 168-ComG-GFP and 168-ComG-GFP containing non-induced pLK. At intermediate levels of IPTG induction (100 µM and 250 µM) a bimodal GFP expression pattern emerges, showing that part of the cell population has initiated competence. At high IPTG concentrations about 40% of the cells express GFP two hours after induction (Fig. 2). Three hours after induction over 90% of the cells express GFP (data not shown), indicating that almost all cells have initiated competence development. This shows that in the laboratory strain, the pLK plasmid system can be used to induce the competence regulon using IPTG induction.

### Inducing competence in environmental strains of *Bacillus subtilis*

Plasmid pLK was introduced via natural competence into strain 168 and by protoplast transformation into strains NCIB3610, ATCC6633 and JW49. In the resulting strains *comK* expression was induced with IPTG as described in the Material and Methods. Cells were subsequently treated with either chromosomal DNA containing a chloramphenicol resistance marker, a replicative plasmid containing a tetracycline resistance marker or an integrating plasmid containing a chloramphenicol resistance marker. After selection on the appropriate selective plates, transformation frequencies were determined by colony counting and the calculated transformation efficiencies are shown in Tables 1 and 2. Transformants resulting from chromosomal DNA or integration vectors that were supposed to recombine at the amylose degradation *amyE* locus were checked for correct integration by testing for amylase activity. Over 90% of the resulting colonies became amylase negative indicating correct integration of the donor DNA.

**Table 1:** Transformation efficiency of tested strains using LB culture medium.

Strain	Transformants/10^6^ cells^a^ at [IPTG]		
	0 µM	200 µM	1000 µM
168 *wt*	0.6	-	-
168-pLK	9.8	16.3	8.7
NCIB3610-pLK	<0.01	-	2.8
JW49-pLK	<0.01	-	0.35

a At the time of plating, the 168 and 168-pLK culture contained 1.3*10^9^ viable cells/ml, the NCIB3610-pLK culture contained 0.9*10^9^ viable cells/ml, and the JW49 contained 1.0*10^9^ viable cells/ml. For transformations 6 µg chromosomal DNA from strain JWV184 per ml culture was used. -  =  not tested.

**Table 2:** Increase in competence levels of tested strains using SMM culture medium.

Strain	[IPTG] µM	Competence induction levelsa,b, with DNA of type:		
		Chromosomal DNA JWV184	Integrative plasmid pGFP-*rrnB*	Replicative plasmid pHY300plk
168-pLK	0	-	-	1.1
168-pLK	200	-	-	1.3
NCIB3610-pLK	0	2.8	3.8	6
NCIB3610-pLK	200	12.8	16.8	20.6
NCIB3610-pLK	1000	20	25	33.3
JW49-pLK	0	4.7	5.5	-
JW49-pLK	200	33.3	50	20

a fold induction of competence compared to the control strain containing the empty pLOSS* vector and grown with 200 µM IPTG.

b In a typical experiment using SMM medium and 200 µM IPTG, the 168-pLK, NCIB 3610-pLK and JW49-pLK cultures contained ∼3.5*10^8^ viable cells/ml at the time of plating (OD_600_ 0.65), and when using pHY300plk plasmid as donor DNA, approximately 40.0*10^4^ (168+ pLK) 3.35*10^4^ (NCIB3610 + pLK) and 0.47*10^4^ (JW49 + pLK) transformants/ml were obtained.

-  =  not tested.

Transformation frequencies were tested in both LB and SMM medium. In LB medium cells normally are poorly competent, and the induction of competence under these conditions demonstrates that the system is able to induce competence under non-competence stimulation conditions. In SMM, cells from the laboratory strain normally do develop competence and this will therefore most likely be the system to optimally assay competence. In the absence of pLK, the *B. subtilis* 168 laboratory strain showed very low (<1 transformant/10^6^ viable cells) levels of natural competence in LB medium. When pLK was introduced and IPTG was added, transformation rates increased up to 27 fold. Competence in the “wild” environmental strains or derivatives of these strains containing the empty vector or pLK without IPTG induction was below detectable limits in LB medium. However, when expression of *comK* was induced from the pLK plasmid, transformants were readily obtained in the environmental strains. The lower level of transformation in 168 at maximum IPTG induction is likely caused by a detrimental effect of ComK overproduction on cell growth [14] (Table 1).

As expected, growing *B. subtilis* 168 in SMM increased natural competence over 3 orders of magnitude. The increase in competence obtained upon IPTG induction of the pLK plasmid in SMM medium was negligible, probably because the culture had already achieved almost optimal competence in SMM without additional *comK* induction. In our laboratory, NCIB3610 typically has a transformation frequency approximately 0.5–1% that of 168 using chromosomal DNA as donor DNA (data not shown) and SMM as the growth medium. However, IPTG induction of strain NCIB3610 containing plasmid pLK increased the frequency of transformation up to 30 fold (Table 2). Also the environmental isolate *B. subtilis* JW49 showed a very low level of competence in SMM, which increased over 30 fold upon induction of the introduced pLK plasmid (Table 2). Interestingly, in SMM the non-induced pLK containing strains showed a 2 to 6 fold increase in transformation frequency compared to the control strains carrying empty plasmid (Table 2). This indicates that *P_spac_* on the pLK plasmid is not completely inactive in the absence of IPTG. In environmental strains growing in LB medium, no effect was observed in the presence of the uninduced plasmid. Under these conditions, the residual expression of *comK* is likely quenched due to the rapid degradation of its product by the MecA-Clp complex [16], or diluted out because of fast growth, and competence does not develop.

### Measuring *comG*-transcription in environmental strains of *Bacillus subtilis*

**Figure 3:**
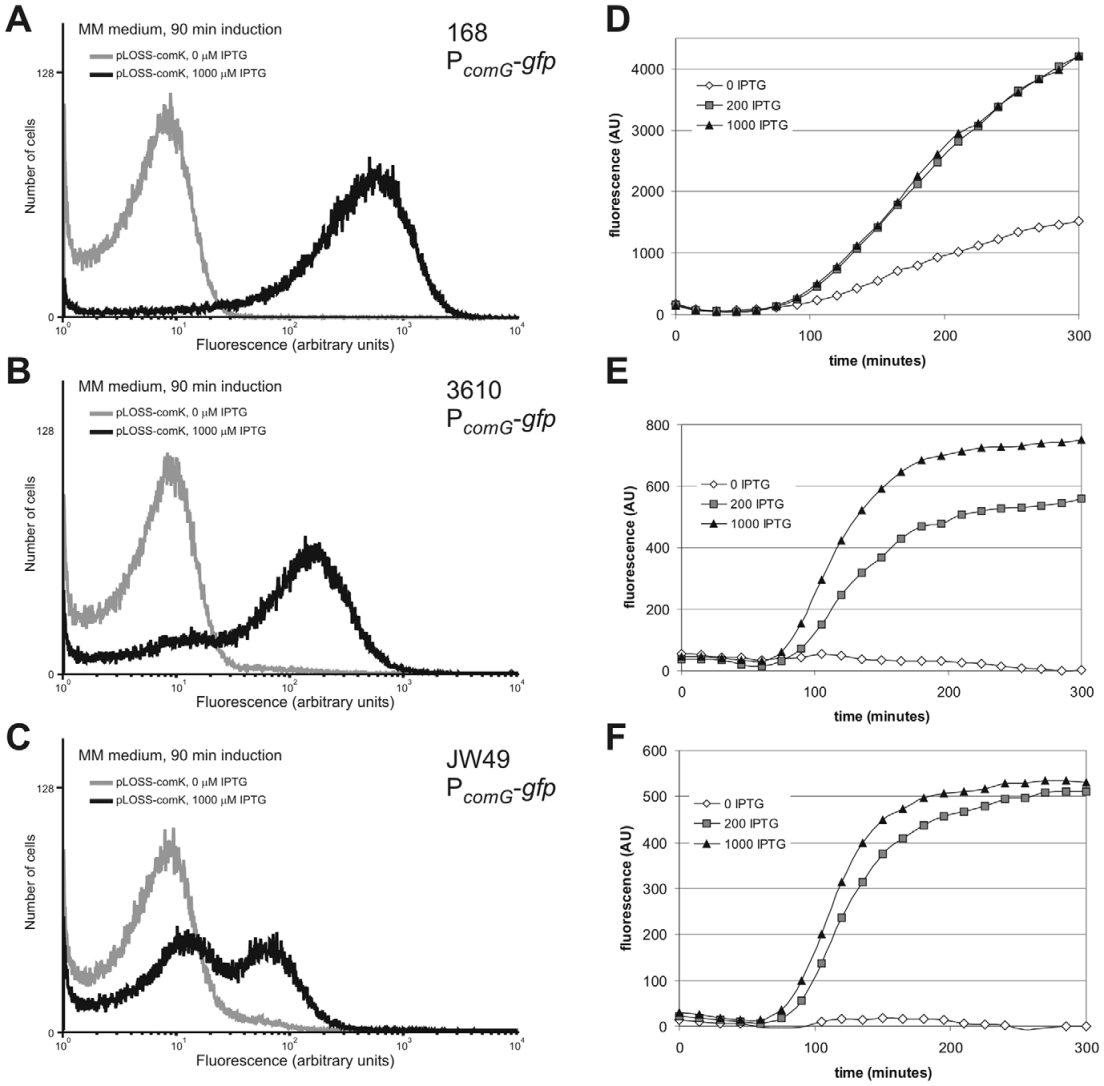
Expression of P*comG*-GFP in environmental *B. subtilis* strains. (**A–C**) Exponentially growing cells (MM medium) were induced for 90 minutes with or without 1000 µM of IPTG. Expression of GFP from the *comG* promoter was analysed by flow cytometry. At least 100.000 cells were analysed for each sample. (**D–F**) P*comG*-GFP expression of the total population after induction with 0, 200 and 1000 µM IPTG, measured using a microtitre plate reader. Note that the timing between data from panels A–C and D–F cannot be directly compared due to the different experimental setups.

We introduced the *P_comG_*-GFP construct in environmental strains NCIB3610 + pLK and JW49 + pLK by transformation with chromosomal DNA of strain 168-ComG-GFP. These strains were made competent using induction of pLK as described above. The fluorescent signal of *P_comG_*-GFP was measured using a microtitreplate reader to test induction with different concentrations of IPTG. Overall levels of *P_comG_*-GFP in these environmental strains were lower compared to *B. subtilis* 168, as expected from the transformation results (Fig. 3D–F). We further analysed these strains using flow cytometry to test whether this difference was due to an overall lower fluorescence or if a smaller part of the population became competent (Fig. 3A–C). The results demonstrate that the levels of competence observed in the environmental strains as determined by transformation assays correlate well with the level of induction observed from the *comG* promoter. At maximum IPTG induction, strain NCIB3610 has not achieved full competence and the green fraction also has a lower overall fluorescence. This effect is even more obvious in strain JW49, where at maximum induction only about half of the cells become highly fluorescent. Also the fluorescence level of the green fraction of this strain is much lower than strains 168 and NCIB3610. When increasing the IPTG levels above a certain threshold concentration (>300 µM, data not shown) no further increase in *comG-gfp* expression was observed. This might suggest that the uptake of IPTG in these environmental strains is lower, or that LacI acts as a better repressor in these strains. More likely however is that *B. subtilis* 168 ComK is not able to fully activate the competence pathway in these environmental strains or that the competence pathway in these strains is less efficiently triggered than in the laboratory strain. It should be noted that the data for the 168 laboratory strain shown in Figs. 2 and 3 cannot be directly compared to each other in terms of fluorescence and timing of *comG-gfp* expression since different growth medium was used (LB vs SMM). We used SMM to analyze the environmental strains since this medium better supports competence than the rich LB medium.

### Plasmid pLK is unstable and can be readily removed from the host

Due to its unstable replicon [15] the pLOSS derived plasmids can be cured from *B. subtilis* strains by incubating at 50°C for 6–8 hours. After curing, the strains are no longer capable of growing on agar containing spectinomycin, and growth on X-Gal does not lead to blue coloration of the colonies. The efficiency of curing the plasmid was tested by plating strain 168+pLK at LB agar containing X-Gal but no spectinomycin, and incubation at 50°C for different times followed by growth over night at 37°C. Incubation at 50°C for 8 hours or more resulted in only white colonies. Incubation for 3 hours resulted in 45% white colonies, and direct transfer to 37°C without the 50°C step resulted in 35% white colonies. When the environmental strains NCIB3610-*P_comG_*-GFP and JW49-*P_comG_*-GFP that originally contained pLK were cured, no fluorescence increase was be observed after induction with IPTG both in the microtitreplate reader assay as when using flow cytometry (data not shown). The flexibility and transient nature of this competence induction system gives it a major advantage over the techniques described until now for *B. subtilis* ATCC6633 [9] or *B. cereus* [10]. Furthermore, by removing the plasmid it also enables other IPTG inducible promoters to be used in the strains of interest.

### ComK overproduction does not induce competence in *Bacillus licheniformis*

Natural competence has been described for *B. licheniformis* [17] although the frequencies are very low [18], [19], [20] or undetectable [21]. We did not succeed in obtaining transformants using competence induction methods developed for both *B. subtilis* [22] or an amended method for *B. licheniformis* [17] to induce natural competence. We tested if competence could be induced in strain *B. licheniformis* DSM13 by induced expression of *comK* as described above. However, with plasmid pHY300plk as donor DNA (a replicative plasmid), no transformants were obtained whether the strain contained pLLK (*B. licheniformis comK*) or pLK (*B. subtilis comK*) (data not shown). Both plasmids pLK and pHY300plk could be successfully introduced in *B. licheniformis* by protoplast transformation, and could also be re-isolated from this strain. This indicates that, although *B. licheniformis* DSM13 contains the core ComK-regulon required for competence development [23], *comK* induction alone is not sufficient to induce competence in this strain under our experimental conditions.

### Concluding remarks

Our method provides a straightforward way of genetically manipulating undomesticated strains such as NCIB3610 and other environmental isolates of *B. subtilis* if they carry a functional *comK* regulon. The pLK plasmid needs to be introduced into the wild strain by e.g. protoplast transformation only once. Subsequently this plasmid-containing strain can be used in multiple transformation experiments. As long as selection for the spectinomycin resistance marker on the plasmid is applied the plasmid will be retained and multiple rounds of transformation can be performed. An added advantage is that competence can be obtained in complex media such as LB, which normally does not support competence. This is beneficial for the transformation of strains that are not able to grow in defined competence media. When the required genetic alteration(s) has been obtained the plasmid can be removed via a simple overnight incubation in the absence of selection, reverting the strain back to its “wild” state.

## Materials and Methods

### Bacterial strains and growth media

Bacterial strains and plasmids used in this study are listed in Table 3. *B. subtilis* and *B. licheniformis* strains were grown at 30 or 37°C under vigorous agitation. The strains were grown in either LB broth (Oxoid, UK) or Spizizen minimal medium (SMM) [22]. For the selection of transformants, appropriate antibiotics (Sigma, UK) were added to the growth media at the following concentrations: chloramphenicol, 5 µg/ml; tetracycline, 6 µg/ml and spectinomycin, 100 µg/ml or 600 µg/ml (if sodium succinate was used in the protoplast regeneration medium). For solid media 1.5% bacteriological agar (Oxoid, UK) was added.

**Table 3:** Strains and plasmids used.

Strains	Relevant properties	Source or reference
***E. coli***		
DH5α		Invitrogen
***B. subtilis***		
168	*trpC2*	[28]
NCIB3610	Undomesticated wild strain	(http://www.bgsc.org/)
ATCC6633	Undomesticated wild strain	[9]
JW49	Isolated from deep sea sediment, mid Atlantic ridge	This study,Genbank #GQ869538
JWV184	168, *amyE*::*P_hyper-spank_*-*gfp*, *Cm^R^*	[29]
168-ComG-GFP	168, *P_comG_*−*gfp*, *CmR*	[27]
NCIB3610-PcomG-GFP	NCIB3610, *P_comG_*−*gfp*, *CmR*	This study
JW-49-PcomG-GFP	JW49, *P_comG_*−*gfp*, *CmR*	This study
*B. licheniformis*		
DSM13		(http://www.bgsc.org/)

Plasmids		
pLOSS*	*ampR, spcR, P_divIVA_-lacZ, pLS20 replicon*	[15]
pLK	pLOSS* containing *B. subtilis comK;*	This study
pLLK	pLOSS* containing *B. licheniformis comK*	This study
pHY300plk	*E. coli- Bacillus* shuttle vector, *tetR*	[30]
pGFP-rrnB	Integrative vector, *amyE*::P*_rrnB_*-*gfp*,*Cm^R^*	[29]

### Strain constructions and transformation

Cloning and transformation procedures were performed according to established techniques [24] and suppliers' manuals. Restriction enzymes, *Taq* DNA polymerase and T4 DNA ligase were obtained from Fermentas Life Sciences (Vilnius, Lithuania), Phusion DNA polymerase was obtained from Finnzymes (Espoo, Finland) and used as specified by the suppliers. Primers were supplied by Invitrogen UK.

### Construction of pLK and pLLK plasmids

To construct the IPTG inducible plasmid pLK (Fig. 1) the *B. subtilis comK* gene was amplified from *B. subtilis* 168 chromosomal DNA by PCR using Phusion DNA polymerase, using primers comK-F+NheI+RBS (GCGCGCTAGCTAAGAGGAGGAACTACTATGAGTCAGAAAACAGACGCACCTTTAGAATCG) and comK-R+term+*Bam*HI (GCGCGGATCCAGGGAGCTTCAATTATCACAACTACC). To construct the *B. licheniformis comK* containing IPTG inducible plasmid pLLK the *B. licheniformis comK* gene was amplified from *B. licheniformis* DSM13 chromosomal DNA using primers Blich-comK-fw+NheI+RBS (GCGCGCTAGCTAAGAGGA GGATCAAAATGAGCACAGAGGATATGACAAAGGATAC) and Blich_comK-rv+bamHI (GCGCGGATCCCGGAAAATCAAGGTGCATGAAGTGTC). Both PCR products were digested with *Nhe*I and *Bam*HI and ligated into the similarly digested vector pLOSS* [15]. The resulting ligation products were transformed into *E. coli* DH5α. Positive colonies were checked by PCR, followed by isolation of the plasmid and restriction check. Selected plasmids where also checked by sequencing (Eurofins, UK) using primers aprE NC-checkF (AAAGTTGTTGACTTTATCTAC) and pLOSS-R-seq (CCTCTGATAGACAGCATGTC). Plasmid pLK has been made available through the Bacillus Genetic Stock Centre, with accession number ECE219.

### Protoplast transformation

The plasmids pLK and pLLK were introduced into *B. subtilis* NCIB3610, ATCC6633, JW49 and *B. licheniformis* DSM13 and EI-34-6 by protoplast transformation [25]. Since this method uses sodium succinate as an osmotic agent, which inhibits spectinomycin activity, the spectinomycin concentration was adjusted to 600 µg/ml [26]. Colonies obtained were checked by colony PCR using primers aprE NC-checkF and pLOSS-R-seq, and subsequently the plasmids were re-isolated from positive clones and checked by restriction with *XbaI* and *BamHI*.

### Flow cytometric measurement of 168 *P_comG_*-GFP fluorescence

To measure induction of competence in *B. subtilis* 168 we used the *P_comG_*-GFP reporter strain 168-ComG-GFP [27]. pLOSS* and pLK were introduced into this laboratory strain using natural competence. Resulting strains were grown as described above, and after addition of IPTG the cells were measured every 30 minutes for individual GFP fluorescence by flow cytometry. Cells were diluted 10-fold in PBS and GFP fluorescence was measured on a Partec CyFlow Space flow cytometer (Partec, Germany) operating a 20 mW solid state laser (488 nm) essentially as described previously [27]. For each sample, at least 100,000 cells were analyzed. Results were visualised using WinMDI 2.9 (http://facs.scripps.edu/software.html).

### Competence assay

To test for induced competence, the strain containing the inducible *comK* plasmid pLK was grown in two different growth media. To test for competence induction in complex, nutrient rich medium, the strains were grown over night in LB with spectinomycin and diluted in LB with spectinomycin to an OD_600_ of 0.2 the next morning. Growth was followed and at an OD_600_ of 1.5, IPTG was added to all cultures in varying concentrations. Cultures were grown for another 45 minutes to allow induction of competence. Induced culture (150 µl) was transferred to a 2 ml microtube containing 5 µl DNA, and the cultures were further agitated at 37°C for 90 minutes. Chromosomal DNA of *B. subtilis* strain JWV184 (180 µg/ml), plasmid pHY300plk (110 µg/ml) and plasmid pGFP-*rrnB* (25 µg/ml) were used. Dilutions were plated on LB agar containing spectinomycin and other appropriate antibiotics. To calculate transformation efficiency, the cultures were also plated on non selective LB agar in a dilution series to establish the viable cell count in the culture. As a control both the strain containing the empty pLOSS* vector and cultures where the IPTG addition was omitted were used. To test competence induction in minimal medium the strains were grown over night in SMM with spectinomycin and diluted 1∶10 the next morning in fresh SMM with spectinomycin, grown for 2–3 h until an OD_600_ of 0.4. IPTG was added to all cultures in varying concentrations and the rest of the protocol was followed as described above.

### *P_comG_*-GFP analysis in environmental strains

Strains 3610+pLK and JW49+pLK were transformed as described above with chromosomal DNA of strain 168-comG-GFP to introduce the competence reporter construct. The resulting strains NCIB3610-*P_comG_*-GFP+pLK and JW49-*P_comG_*-GFP+pLK were grown overnight in SMM medium containing appropriate antibiotics. Flow cytometric analysis was performed as described above. For analysis using the microtitreplate-reader, the strains were diluted 1∶3 in fresh SMM medium and split in 3 separate aliquots: no IPTG added, 200 µM IPTG added, 1000 µM IPTG added. These cultures were transferred to a clear 96 well flat bottom polystyrene tissue culture plates (BD-Falcon, USA) using 200 µl culture/well. The plate was grown in a Fluostar Optima plate reader (BMG labtech, UK) at 37°C with constant double orbital shaking (150 rpm/4 mm orbit) in between measurements. Both the absorbance at 595 nm (filter A595) and the fluorescence (excitation 485 nm/emission 520 nm (filters 485-12 and EM520)) were measured every 15 minutes for each well. The fluorescence signal from 4 identical wells was averaged and corrected for the GFP-negative strain that was grown in the same plate at the same OD_595_ (3610+pLK and JW49+pLK).

### Curing of the pLOSS-comK plasmid

To cure the strains of the pLK plasmid the method described by Claessen *et al.* [15] was used. Briefly, after the required genetic manipulation, the pLK containing transformed strains were plated on LB agar containing X-gal without spectinomycin and incubated at 50°C for 6–8 hours, after which they were incubated over night at 37°C. Resultant white colonies were apparently cured of the plasmid, which was confirmed by checking for spectinomycin sensitivity by re-streaking on LB containing spectinomycin and X-gal. Furthermore, we checked for the loss of the ability to induce competence as measured by *P_comG_*-GFP fluorescence upon induction with IPTG, using the microtitreplate reader as described above.
